# Integrating Molecular Insights into Biliary Tract Cancer Management: A Review of Personalized Therapeutic Strategies

**DOI:** 10.3390/curroncol31070266

**Published:** 2024-06-21

**Authors:** Mar Ros-Buxó, Ezequiel Mauro, Tamara Sauri, Gemma Iserte, Carla Fuster-Anglada, Alba Díaz, Laura Sererols-Viñas, Silvia Affo, Alejandro Forner

**Affiliations:** 1 School of Medicine, Universitat de Barcelona, 08007 Barcelona, Spain; mrosbuxo@gmail.com (M.R.-B.); sauri@clinic.cat (T.S.); madiaz@clinic.cat (A.D.); 2 Institut d’Investigacions Biomèdiques August Pi i Sunyer (IDIBAPS), 08036 Barcelona, Spain; giserte@clinic.cat (G.I.); fuster@clinic.cat (C.F.-A.); lsererols@recerca.clinic.cat (L.S.-V.); saffo@recerca.clinic.cat (S.A.); 3 Centro de Investigación Biomédica en Red de Enfermedades Hepáticas y Digestivas (CIBERehd), 28029 Madrid, Spain; 4 Barcelona Clinic Liver Cancer (BCLC) Group, Liver Unit, Institut Clínic de Malalties Digestives i Metabòliques (ICMDM), Hospital Clinic Barcelona, 08036 Barcelona, Spain; 5 Barcelona Clinic Liver Cancer (BCLC) Group, Medical Oncology Department, Institut del Càncer i Malalties de la Sang (ICAMS), Hospital Clinic Barcelona, Translational Genomics and Targeted Therapies in Solid Tumors, IDIBAPS, 08036 Barcelona, Spain; 6 Barcelona Clinic Liver Cancer (BCLC) Group, Pathology Department, CDB, Hospital Clinic Barcelona, 08036 Barcelona, Spain

**Keywords:** BTC, cholangiocarcinoma, molecular profile, ESCAT, FGFR2, IDH1, BRAF, KRAS, NTRK, liver transplantation

## Abstract

Biliary tract cancers (BTCs) are rare and aggressive malignancies with an increasing incidence and poor prognosis. The standard systemic treatment for BTCs has evolved to include immune checkpoint inhibitors associated with gemcitabine–cisplatin as first-line therapies. However, survival rates remain low, highlighting the critical need for personalized treatment strategies based on molecular profiling. Currently, significant advancements have been made in the molecular characterization of BTCs, where genetic alterations, such as *IDH1* mutations and *FGFR2* fusions, provide targets for therapy. Molecular profiling is crucial early in the management process to identify potential candidates for clinical trials and guide treatment strategy. The integration of these molecular insights into clinical practice has allowed for the development of targeted therapies, although many of them are still in the phase 2 trial stage without definitive survival benefits demonstrated in phase 3 trials. This integration of comprehensive molecular profile insights with traditional treatment approaches offers a new horizon in the personalized medicine landscape for BTCs, with the aim of significantly improving patient outcomes through precision oncology.

## 1. Introduction

Biliary tract cancers (BTCs), including gallbladder carcinoma (GBC), cholangiocarcinoma (CCA), and ampullary adenocarcinoma, constitute a rare and aggressive group of malignancies [1]. These cancers account for 2% of digestive system cancers and 10–15% of primary liver cancers, and their incidence has been increasing in recent years [2]. The principal risk factors include cholelithiasis, biliary flukes in Asia, chronic inflammatory diseases of the bile ducts, metabolic syndrome-associated liver diseases, such as metabolic dysfunction-associated steatotic liver disease (MASLD), tobacco use, chronic hepatitis B and C infections, and cirrhosis [3,4].

BTCs are notably heterogeneous and are typically classified by their primary anatomical origin: (1) CCA, subdivided into intrahepatic (iCCA), accounting for 10–20% and arising from second-order intrahepatic bile ducts; perihilar (pCCA), which accounts for 50% and originates from the right, left, and/or common hepatic duct; and distal (dCCA), constituting 30–40%, which develops from the common bile duct below the cystic duct insertion; (2) GBC; and (3) ampullary adenocarcinoma. This diversity extends beyond anatomy to include various genomic alterations that are distributed disparately across BTC subtypes, which also influences its prognosis [1].

The incidence of iCCA has been increasing, in contrast to the stable rates of extrahepatic forms, driven largely by an increase in chronic liver conditions, the wide use of percutaneous biopsies for studying liver nodules, and more awareness of this disease, mainly by pathologists [5,6]. Over the past three decades, the prognosis for BTCs has remained poor, with relative survival rates of 1, 3, and 5 years post-diagnosis estimated at 25%, 10%, and 7%, respectively. Approximately 65% of patients receive only the best supportive care at the time of diagnosis. Even in the early stages, where surgery is the treatment of choice, 5-year overall survival (OS) rarely exceeds 5–10% in GBC (except for very early stages where 5-year OS reaches 95–100%) and 12–40% in CCA [7,8,9].

Historically, the standard treatment for locally advanced or metastatic cases was based on the combination of gemcitabine and cisplatin (GEMCIS), as demonstrated by the ABC-02 study, which showed a median OS (mOS) of 11.7 months for GEMCIS versus 8.1 months for gemcitabine alone [10]. The treatment landscape has evolved with the addition of immune checkpoint inhibitors (ICIs), such as durvalumab [11] or pembrolizumab [12], which have demonstrated further survival benefits over GEMCIS alone. These triple combinations are now recommended as first-line therapies according to various guidelines, including the ESMO, EASL-ILCA, ASCO, and national guidelines in the UK and France [8,13,14,15]. Additionally, the ABC-06 study established leucovorin, 5-fluorouracil, and oxaliplatin (FOLFOX) as standard-of-care chemotherapy in the second-line setting, showing significant benefits over placebo (6.2 vs. 5.3 months; HR 0.69, 95% CI 0.50–0.97; *p* = 0.031) [16].

Significant advances in our understanding of the molecular biology of BTCs have facilitated the development of numerous effective targeted therapies [17]. Although molecular profiling has been extensively researched and has introduced a variety of new therapeutic options for BTCs, existing data are predominantly from non-randomized studies. Moreover, the effectiveness of these therapies is frequently limited by the genetic heterogeneity inherent in BTCs and the adaptability of therapeutic targets [18]. Given these challenges, there is a compelling need for future strategies that integrate immunotherapy, chemotherapy, and targeted treatments to deliver sustained clinical benefits and enhance survival. This review summarizes the latest evidence on molecular profiling and personalized medicine for BTC management.

## 2. Molecular Profiling in the Landscape of Biliary Tract Cancers

While we currently lack phase 3 trials with targeted therapies for locally advanced or metastatic BTCs that have demonstrated an OS benefit, the genetic richness of BTCs, particularly iCCA, in potentially druggable tumor gene alterations and the clinical success observed with various targeted therapies in phase 2 studies (some already approved and marketed) underscore the role of systematically assessing the molecular profile of BTCs [19]. Table 1 summarizes the main actionable alterations.

The guidelines recommend molecular profiling of the tumor during the first line of treatment, recognizing that oncogenic driver alterations occur early during tumorigenesis and persist throughout the course of the disease without significant changes in the absence of selection by targeted therapy [8,13,20]. Tumor molecular profiling should detect gene fusions/rearrangements, ideally through RNA next-generation sequencing (NGS) and mutations of therapeutic interest according to the ESMO Scale for Clinical Actionability of Molecular Targets (ESCAT) [21,22,23]. Moreover, in addition to molecular profiling, it is crucial to systematically investigate the microsatellite instability/mismatch repair deficiency (MSI/dMMR) status of the tumor through immunohistochemistry (IHC) and/or polymerase chain reaction (PCR) testing, taking into account the potential benefits of immunotherapy and the possibility of human epidermal growth factor receptor 2 (HER2) overexpression/amplification, which can be assessed through IHC and/or in situ hybridization (ISH).

### 2.1. Isocitrate Dehydrogenase (IDH-1)

IDH-1 is a crucial enzyme in cellular metabolism that catalyzes the conversion of isocitrate to α-ketoglutarate. Specific gain-of-function mutations in *IDH-1* lead to the production of D-2-hydroxyglutarate, a metabolite implicated in the pathogenesis of various cancers, including glioma, acute myeloid leukemia, and iCCA, which is found in 13–25% of cases [24]. Identification of *IDH-1* mutations in iCCA is vital for prognosis and enables the development of targeted therapeutic strategies [25].

Ivosidenib, an inhibitor targeting the mutated IDH-1 enzyme, was initially approved for the treatment of newly diagnosed acute myeloid leukemia. In the realm of iCCA, the ClarIDHy study stands out as the sole phase 3 randomized trial targeting this mutation [26]. This study showed that ivosidenib significantly improved progression-free survival (PFS) compared to placebo (median: 2.7 vs. 1.4 months; HR: 0.37 [95% CI 0.25–0.54]; *p* < 0.001) in 185 patients with advanced iCCA and *IDH-1* mutations who had progressed after one or two prior chemotherapy lines, maintaining an ECOG-PS of 0–1. Although initial improvements in OS were not apparent, significant enhancements in OS were observed after adjusting for the 71% of patients who crossed over to ivosidenib following progression in the placebo group (mOS: 10.3 vs. 5.1 months; HR: 0.49; *p* < 0.001) [27]. Common all-grade adverse events (AEs) included nausea (41%), diarrhea (35%), and fatigue (31%). The rates of severe (grade ≥ 3) AEs were similar between the ivosidenib (7%) and placebo (9%) arms, with discontinuation due to AEs occurring in 7% of the patients in the ivosidenib group and none in the placebo group. Notably, those treated with ivosidenib maintained their quality of life compared to those receiving placebo. Following the promising results from the ClarIDHy trial, both the FDA and EMA approved ivosidenib for use in adult patients with previously treated, locally advanced, or metastatic *IDH-1*-mutated CCA.

### 2.2. Fibroblast Growth Factor Receptor (FGFR)

The FGFR family, which encompasses several receptors, plays a pivotal role in essential cellular processes, such as proliferation, differentiation, migration, and survival. Specifically, FGFR2 interacts with distinct FGF ligands to regulate cellular signaling pathways, including the MAPK and PI3K/AKT pathways. Genetic aberrations in *FGFR2*, such as amplifications, point mutations, or fusions, are implicated in unchecked cellular proliferation, notably in ~15% of iCCA cases [24]. Consequently, targeted therapies against FGFR2, particularly competitive, reversible ATP inhibitors, have shown considerable promise [28].

Several non-randomized phase 1/2 or 2 studies have confirmed the efficacy of oral pan-FGFR or FGFR2 inhibitors in patients with advanced CCA harboring *FGFR2* fusions or rearrangements who had previously undergone at least one line of systemic therapy [29,30,31,32,33]. Notably, pemigatinib and infigratinib, selective but reversible inhibitors of FGFR1-3, bind competitively to the ATP pocket within the kinase domain and have been effectively used to treat iCCA with positive *FGFR2* fusion/rearrangement [34].

Futibatinib, a selective inhibitor of FGFR1-4, differentiates itself by covalently binding to a cysteine residue within the FGFR kinase domain, which suppresses the FGFR signaling cascade, inhibits tumor cell proliferation, and induces cell death. This covalent and irreversible attachment to the ATP pocket renders futibatinib less susceptible to resistant mutations. Futibatinib has demonstrated antiproliferative activity in preclinical studies by targeting a broad spectrum of FGFR aberrations [34].

In clinical settings, pemigatinib and futibatinib exhibited ORR of 35.5% and 42%, median PFS (mPFS) of 6.9 and 9.0 months, and mOS of 21.1 and 21.7 months, respectively, in the single-arm phase 2 trials FIGHT-202 and FOENIX-CCA2 [35,36]. These trials included 107 and 103 patients with FGFR2-rearranged iCCA naive to FGFR inhibitors, respectively. Hyperphosphatemia, typically of low severity, was the most common adverse effect associated with both and was managed with dose adjustments or interruptions as needed. Despite its distinct mechanism, the efficacy of futibatinib in tumors that are resistant to conventional FGFR inhibitors has been questioned. The FOENIX-CCA2 trial excluded patients previously treated with FGFR inhibitors owing to potential resistance. The administration of futibatinib resulted in tumor regression and CA19-9 level reduction in cases with mutations, such as V565L and N549D, which are resistant to pemigatinib. Both pemigatinib and futibatinib have received FDA and EMA approval for use in patients with previously treated locally advanced or metastatic CCA, highlighting their potential as part of the therapeutic arsenal against this challenging malignancy [8,13].

The latest additions to FGFR inhibitors are RLY-4008 [37] and tinengotinib [38]. RLY-4008 is a highly selective irreversible FGFR2 inhibitor that preferentially binds to this receptor in the FGFR family. Preliminary findings from three cases in an ongoing phase 1/2 study (NCT04526106) have demonstrated significant responses in patients with CCA who have not been previously treated with FGFRi and exhibit FGFR2 aberrations. The unique selectivity of RLY-4008 distinguishes it from other inhibitors because of the structural similarities among FGFR2 family members, offering a new approach to overcoming the resistance seen in patients treated with pan-FGFR inhibitors. In contrast, futibatinib covalently and irreversibly inhibits all FGFR receptors. The specificity provided by RLY-4008 is also instrumental in combating resistance, which is a significant challenge for current therapies. Researchers have explored conformational differences between FGFR2 and other FGF receptors to identify new therapeutic targets, including oncogenic resistance mutations in FGFR2. In addition to RLY-4008, tinengotinib may offer promising clinical benefits for patients with CCA, particularly in the context of resistance to FGFRi. This multi-kinase inhibitor has a unique binding mechanism to FGFR that not only targets FGFR2 fusions and rearrangements but also acquires resistant mutations. The efficacy and safety of tinengotinib were evaluated in a phase 2 trial in which the primary endpoint was the ORR. Among the 15 patients with FGFR alterations, over 93.3% had previously been treated with more than one FGFRi and 3 patients had received two FGFRi treatments. The trial reported an ORR of 34% and mPFS of 6.9 months [38]. Preliminary biomarker analysis suggested a reduction in resistant *FGFR* mutations in liquid biopsies following tinengotinib treatment. This ongoing phase 2 trial will provide crucial data on safety, efficacy, and biomarker assessment for iCCA resistant to FGFR inhibitors. Table 2 summarizes the main trials evaluating agents targeting FGFR.

Finally, regarding the two in-progress phase 3 trials, the FIGHT-302 (NCT03656536), comparing first-line pemigatinib versus GEMCIS in the first line, was prematurely interrupted due to low recruitment. Accordingly, the FIRST-308 trial (NCT05948475), comparing tinengotinib with the investigator’s choice of standard chemotherapy in patients with FGFR2-altered iCCA refractory to FGFR inhibitors, is the only phase 3 trial currently ongoing.

### 2.3. Human Epidermal Growth Factor Receptor 2 (HER2)

The HER2 is a tyrosine kinase receptor that belongs to the epidermal growth factor receptor family and plays a pivotal role by activating the PI3K-AKT-mTOR signaling pathway. Amplifications, overexpression, and, more rarely, mutations of *HER2* lead to constitutive activation of the growth factor cascade, thereby acting as an oncogenic driver in various cancers. These alterations are observed in approximately 15% of BTCs, predominantly GBC, pCCA, dCCA, and ampullary adenocarcinoma [24,34].

Trastuzumab, a monoclonal antibody, has shown clinical benefits in both the metastatic and adjuvant settings. Zanidatamab, a bispecific antibody, targets two distinct epitopes of HER2: the extracellular domain and the dimerization domain. Although trastuzumab and pertuzumab target these two epitopes, zanidatamab exhibits stronger antitumor activity and significant clinical effects, even in tumors with low HER2 expression.

Several non-randomized phase 1/2 trials involving patients with chemorefractory, *HER2*-overexpressed/amplified advanced BTCs have demonstrated an ORR ranging from 23% to 47%, mPFS from 4.0 to 5.5 months, and mOS from 7.1 to 10.9 months [7,19]. These trials tested various HER2 inhibition strategies, including combinations of the anti-HER2 monoclonal antibodies trastuzumab and pertuzumab [40], the HER2 antibody–drug conjugate trastuzumab with modified FOLFOX [39], bispecific anti-HER2 antibody zanidatamab [41], and combinations of trastuzumab and tucatinib [42]. Table 2 summarizes the main trials based on HER2. The use of HER2 inhibitors in the first-line setting is in development. In a single-arm, phase 2 trial, 90 chemonaïve BTC patients treated with a frontline combination of trastuzumab and GEMCIS achieved an ORR of 55.5% and mPFS of 7 months [46]. The median follow-up period for this trial was 17.3 months, although the mOS was not specified. Additionally, the ongoing phase 3 trial HERIZON-BTC (NCT04466891) evaluates the potential benefits of adding zanidatamab to first-line GEMCIS–durvalumab in improving treatment outcomes for patients with BTCs.

### 2.4. Microsatellite Instability and Mismatch Repair Deficient (MSI/dMMR)

Errors, often referred to as mismatches in the context of DNA replication, typically occur within repetitive DNA sequences known as microsatellites, leading to a condition known as microsatellite instability (MSI). The proteins responsible for correcting these mismatches, including MLH1, MSH2, MSH6, and PMS2, tend to lose their functionality, resulting in the accumulation of mutations within the cell. Although these mismatch repair-deficient (dMMR) tumors may arise in genetic syndromes such as Lynch syndrome, they more commonly emerge as sporadic cases where one allele is spontaneously mutated and the other is epigenetically silenced. These tumors frequently exhibit lymphocyte infiltration due to their high mutation burden and may express PD-L1 on their cellular membranes, making them susceptible to immune system attacks.

Approximately 2% of BTCs exhibit MSI/dMMR, whether constitutional (as in Lynch syndrome) or acquired (sporadic) [24]. Although GEMCIS in combination with pembrolizumab has been established as a first-line treatment for BTCs [12], in the presence of MSI/dMMR-positive BTCs, pembrolizumab has also been investigated as a second-line therapy [7]. A non-randomized phase 2 trial of immunotherapy with pembrolizumab included 22 previously treated advanced BTC patients with MSI/dMMR who had an ECOG-PS of 0–1 [43]. This trial showed an ORR of 40.9%, which is similar to the 34.3% ORR observed in the overall trial population of 233 patients with advanced non-colorectal MSI/dMMR cancers [43]. Currently, pembrolizumab is approved by the FDA and EMA for the treatment of MSI/dMMR BTCs.

### 2.5. BRAF/MEK Inhibitors

V-Raf murine sarcoma viral oncogene homolog B1 (BRAF) and MEK are two critical oncogenic proteins within the MAPK signal transduction cascade, and their activating mutations are prevalent in a wide array of cancers including melanoma and colorectal cancer. Specific therapies targeting the most common *BRAF* mutation, V600E, are in use, although this mutation is found in less than 5% of CCA cases, especially iCCA [24]. 

The EAY131-H open-label, single-arm trial evaluated the combination of BRAF and MEK1/2 inhibitors, dabrafenib and trametinib, in patients whose tumors harbored a *BRAF* V600E mutation and had progressed on at least one standard therapy. Although no complete response was reported, durable partial responses were observed. Among the four iCCA patients out of 35 recruited for the study, three demonstrated significant partial responses lasting 12.8, 9.1, and 29.4 months, with an overall disease control rate of 75.9%. Dabrafenib and trametinib showed an ORR of 38% in the pretreated cohort [44].

In the multicenter basket trial ROAR, patients over 18 years of age with *BRAF* V600E-mutated, unresectable, metastatic, locally advanced, or recurrent BTCs, who had received previous systemic treatment, were recruited and treated with dabrafenib and trametinib. After 10 months of follow-up, the ORR, mPFS, and mOS were 58.1%, 9.0, and 13.5 months. Although no treatment-related deaths were reported, 40% of the patients experienced serious adverse events, and 21% had serious treatment-related adverse events. The most common grade 3 AEs were fatigue, neutropenia, hyponatremia, and hypophosphatemia, with one patient reporting grade 4 sepsis and no grade 5 AEs [45,47]. This body of evidence, particularly from the ROAR trial, led to agnostic FDA approval of dabrafenib and trametinib in this population. However, no approval was granted by the EMA.

### 2.6. Kirsten Rat Sarcoma Viral Oncogene Homolog (KRAS)

The *KRAS* mutations are detected in about 20% of BTCs, with KRASG12C mutations comprising approximately 1% of BTCs and representing the primary actionable target [19,24]. In a phase 1/2 trial, the selective KRASG12C inhibitor adagrasib demonstrated notable efficacy in a cohort that included 12 BTC patients, achieving an ORR of 47.1%, mPFS of 8.6 months, and mOS of 15.1 months [52]. However, there is currently no reported activity data for another KRASG12C inhibitor in BTCs.

### 2.7. Neurotrophic Tropomyosin Receptor Tyrosine Kinase (NTRK)

The *NTRK* gene fusions involving NTRK1, NTRK2, or NTRK3 are rare in BTCs, with a prevalence of <1% [48]. Clinical trials with the oral NTRK inhibitors larotrectinib and entrectinib have demonstrated efficacy; larotrectinib achieved a 75% ORR in a phase 1–2 study across 17 cancer types, including an ORR in one of two CCA patients [49]. Similarly, entrectinib showed a 57% ORR in a pooled analysis of three phase 1–2 trials, which included one CCA patient [53]. Both drugs have received approval from the FDA and EMA for the treatment of solid tumors with NTRK fusions.

### 2.8. Rearranged during Transfection (RET)

RET gene fusions, which are found in various malignancies, have been identified in approximately 1% of BTC cases [50]. In phase I/II trials, the oral RET inhibitors pralsetinib and selpercatinib showed clinical efficacy in patients with RET fusion-positive solid tumors, including BTCs. Specifically, pralsetinib [51] and selpercatinib [54] achieved an ORR of 57% and 43.9%, a mPFS of 7.0 and 13.2 months, and a mOS of 14.0 and 18.0 months, respectively. Currently, these agents have been approved by the EMA for use in RET fusion-positive advanced non-small cell lung cancer and thyroid cancers. Table 3 summarizes the main side effects associated with target therapies.

## 3. Integrating Molecular Profiling into Clinical Practice for BTC Management

The integration of molecular profiles into clinical practice has marked a significant shift in the management of BTCs. Systematic molecular profiling of the tumor should be conducted as soon as the diagnosis is confirmed, even before the initiation of first-line treatment, in order to plan the best treatment sequence and to evaluate potential clinical trial enrollment [8,13]. Recommended assessments include MMR status (by IHC and/or PCR), *HER2* (by IHC; ISH if IHC is 2+), and NGS panels (DNA or RNA) that include the search for actionable tumor mutations (e.g., *FGFR2*, *IDH1*, *BRAF*, *KRAS*, and *NTRK*) [14,23]. While there is no established consensus, molecular profiling of circulating tumor DNA should be considered when tumor tissue is unavailable, and a new biopsy is impracticable. In cases of multiple molecular alterations, it is imperative to discuss the molecular results within multidisciplinary teams.

**Figure 1:**
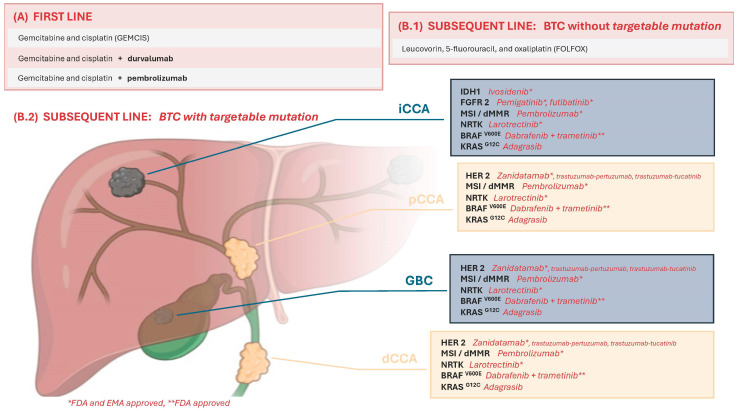
Graphical representation of targeted therapies for BTC. BTC: biliary tract cancer; iCCA: intrahepatic cholangiocarcinoma; pCCA: perihilar cholangiocarcinoma; GBC: gallbladder cancer; dCCA: distal cholangiocarcinoma; FDA: Food and Drug Administration; and EMA: European Medicines Agency.

It is important to note that the current indication for these treatments predominantly occurs in the second-line setting for patients with an ECOG-PS of 0–1, preserved liver function, and the presence of ESCAT levels I-II [8,13,14]. These are grade B recommendations according to the GRADE scale, except for those targeting IDH-1, which, supported by phase 3 studies, could be considered grade A (Figure 1).

## 4. New Horizons in Neoadjuvant Therapy

Comprehensive NGS not only aids in selecting potentially druggable profiles but also reveals significant prognostic implications for certain genetic alterations [23]. *TP53*, *KRAS*, and *CDKN2A* mutations have been identified as independent predictors of worse overall survival, highlighting the critical role of molecular profiling in predicting outcomes for iCCA [55]. These findings underscore the importance of incorporating genetic profiling into the therapeutic decision-making process, especially in potential contexts, such as liver transplantation (LT) for iCCA.

Neoadjuvant therapy, although well established for pCCA, has been less explored in iCCA prior to LT. A prospective study by Lunsford et al. treated 12 patients with locally advanced, unresectable iCCA with gemcitabine-based chemotherapy, six of whom underwent LT, achieving a notable five-year survival rate of 83.3% [56]. Another study by McMillan et al. included 37 patients with unresectable iCCA, all of whom received similar neoadjuvant therapy. Impressively, this approach made surgery an option for five patients. Of the 32 patients who continued to LT, 18 demonstrated disease stability for at least six months, resulting in a five-year survival rate of 57% among transplant recipients, which is in stark contrast to 0% among non-transplant recipients [57].

As the landscape of oncologic therapy evolves, novel targeted therapies in advanced stages have shown promising response rates, indicating their potential expansion into neoadjuvant applications. This could significantly affect peritransplant outcomes in patients with iCCA. Conversely, the use of ICIs along with chemotherapy before LT is constrained by concerns about allograft rejection. Ongoing clinical trials are assessing the efficacy of neoadjuvant treatments in this context, with results expected in the coming years (NCT04195503, NCT06098547, NCT06140134, and NCT04556214).

It is crucial to note that advancements in the field of BTCs suggest that integrating genetic profiling into pre-treatment therapeutic decision-making and combining clinical variables with targeted tumor sequencing may identify patient subgroups with poor outcomes, regardless of the treatment strategy.

## 5. Limitations in the Use of Molecular Profiling

While molecular profiling has significantly enhanced our understanding of BTCs and facilitated the development of targeted therapies, it has limitations. One major shortcoming is that it often overlooks the complexity and heterogeneity of these tumors, which can result in incomplete cancer characterization [58]. Moreover, molecular profiling typically focuses on genetic mutations and may miss important epigenetic changes, protein expression, and tumor microenvironment factors that play crucial roles in cancer characterization and treatment response [59].

In addition to molecular profiling, epigenetic modifications offer a promising avenue for personalized medicine in BTCs. Epigenetic changes, such as DNA methylation and histone modifications, are instrumental in the regulation of gene expression and can contribute to cancer development and progression. Targeting these modifications provides an alternative therapeutic strategy. For example, EZH2 inhibitors can disrupt histone methylation and have shown potential in preclinical models. Similarly, DNA methylation inhibitors can reactivate silenced tumor suppressor genes, thereby offering another therapeutic option. By integrating epigenetic profiling with molecular data, we could potentially achieve a more comprehensive understanding of cancer in each patient, leading to more precise and effective treatment plans [59].

Also, we note that there are practical challenges, such as high costs, limited availability, and variability in the quality of assessment techniques, which are key points for the entry of clinical practice. Finally, most of these targeted therapies are expensive, and despite FDA and EMA approvals and growing evidence of effectiveness, they are not widely available.

## 6. Conclusions

Molecular profiling has markedly broadened the array of targeted therapies available for BTCs, thereby enhancing personalized treatment approaches. However, much of the supportive evidence for these therapies is derived from non-randomized studies, with the notable exception of the CLARIDHY trial, which specifically addressed *IDH-1*-mutated CCA. Conducting randomized trials in first-line settings is challenging due to the rarity of molecularly distinct BTC subpopulations and the lengthy process required for comprehensive molecular profiling. An example of an effort to overcome these hurdles is the PRODIGE 80 SAFIR-ABC10 phase 3 trial (NCT05615818), which aimed to assess the feasibility and benefits of early molecular profiling. This trial seeks to guide maintenance therapy with targeted agents for patients showing a response or stability after GEMCIS–durvalumab treatment, aiming to integrate precision medicine into standard care and potentially improve treatment outcomes in BTCs.

Furthermore, the promising ORR observed in the advanced stages suggests new possibilities for the role of neoadjuvant therapy and its impact on prognosis, particularly in the context of iCCA and LT. These advancements forecast a transformative era in the management of BTCs, highlighting the increasing importance of molecular profiling in shaping the future therapeutic landscape.

## Figures and Tables

**Table 1:** Key actionable alterations.

Mutation	Frequency in CCA	ESCAT
*IDH-1* mutation	1–18% iCCA: 8–18%	I A
*FGFR2* fusion/rearrangement	<10% iCCA: 5–15%	I B
*HER2* overexpression/amplification	5–10% pCCA/dCCA/GBC: 10–20%	I C
MSI/dMMR	<1%	I C
*BRAF* V600E	1–5%	II B
*NRTK* fusion	<1%	I C
*KRAS* mutation	<1%	II B
*RET* fusion	1%	I C

**Table 2:** Clinical trials evaluating targeted therapy in BTCs.

Target	Trial	Drug	Phase	Cohort	Patient Number	Primary Endpoint	ORR	mPFS Months	mOS Months
**FGFR2 fusion/rearrangement**	FIGHT-202 [35]	**Pemigatinib**	II	FGFR2 fusion/rearrangement	108	ORR	37%	7.0 (6.1–10.5)	17.5 (14.4–22.9)
	FOENIX-CCA2 [36]	**Futibatinib**	II	FGFR2 fusion/rearrangement	103	ORR	41.7%	9.0 (6.9–13.1)	21.7 (14.5–NR)
	ReFocus [37]	**RLY-4008**	I/II	FGFR fusion/rearrangement or other alterations in FGFRi-naive CCA	38	ORR	53%	6.9	-
**IDH-1 mutation**	ClarIDHy [25,26]	**Ivosidenib**	III	CCA with IDH-1 mutation and progression on prior therapy	230	PFS	2%	2.7 (1.4 on placebo arm)	10.3 (5.1 on placebo arm)
**HER2 overexpression/amplification**	My Pathway [38]	**Pertuzumab plus trastuzumab**	II a b Basket	Previously treated BTCs with HER2 amplification/overexpression	11 BTCs 8 amplifications, 3 mutations	ORR	3/8 1/3	4.2 2.8	-
	HERIZON-BTC-01 [39]	**Zanidatimab**	II b	Previously treated BTCs with HER2 amplification/overexpression	80	ORR	41.3%	5.5	-
	KCSG-HB19-14 [40]	**Trastuzumab plus modified FOLFOX**	II	HER2+ BTCs and GEMCIS progression	34	ORR	29.4%	5.1	-
	SGNTUC-019 [41]	**Trastuzumab plus** **tucatinib**	II	Previously treated HER2+ metastatic BTCs with no prior HER2-directed therapy	30	ORR	46.7%	5.5	53.6
	TAB [42]	**Trastuzumab plus GEMCIS**	II	HER2+ treatment-naïve BTCs	90	ORR	55.5%	7	-
**BRAF V600E**	EAY131-H [43]	**Dabrafenib** **plus trametinib**		BRAF V600E patients on progression	4	ORR	38%	11.4	28.6
	ROAR [44,45]		II	BTCs with BRAF V600E	43	PFS	42%	9	13.5
**MSI/dMMR**	KEYNOTE-158 [46]	**Pembrolizumab**	II	Previously treated advanced BTC patients with MSI/dMMR	22	ORR	40.9%	4.2	24.3
**KRAS**	KRYSTAL-1 [47]	**Adagrasib**	II	KRASG12C-mutated advanced solid tumors	12	ORR	47.1%	8.6	15.1
**NTRK**		**Larotrectinib** [48]	I/II	TRK fusion + patients	55	ORR	75%	-	-
	ALKA-372-001 STARTRK-1 STARTRK-2	**Entrectinib** [49]	I/II	Metastatic or locally advanced *NTRK* fusion + solid tumors	54	ORR and mDR	57%	11.2	21
**RET**	ARROW [50]	**Pralsetinib**	I/II	RET fusion + solid tumor types	29	ORR	57%	7	14
	LIBRETTO-001 [51]	**Selpercatinib**	I/II	RET fusion + non-lung or thyroid advanced solid tumors on progression on or after previous systemic therapy	45	ORR	43.9%	13.2	186

**Table 3:** Main side effects associated with target therapies.

Target Therapy	Main Side Effects
IDH-1 mutation inhibitors	Nausea, diarrhea, leukocytosis, and fatigue
FGFR2 fusion/rearrangement inhibitors	Hyperphosphatemia, fatigue, dry mouth, and alopecia
HER2 inhibitors	Cardiotoxicity, diarrhea, and nausea
MSI/dMMR inhibitors	Fatigue, rash, pruritus, and immune-related adverse events
BRAF inhibitors	Arthralgia, rash, fatigue, and nausea
NTRK fusion inhibitors	Dizziness, fatigue, constipation, and anemia
KRAS mutation inhibitors	Nausea, diarrhea, hepatotoxicity, and visual disturbances
RET fusion inhibitors	Hypertension, diarrhea, elevated liver enzymes, and dry mouth
